# Multiple signatures of a disease in potential biomarker space: Getting the signatures consensus and identification of novel biomarkers

**DOI:** 10.1186/1471-2164-16-S7-S2

**Published:** 2015-06-11

**Authors:** Ghim Siong Ow, Vladimir A Kuznetsov

**Affiliations:** 1 Bioinformatics Institute, Agency for Science, Technology and Research (A*STAR), Singapore; 2 School of Computer Engineering, Nanyang Technological University (NTU), Singapore

## Abstract

**Background:**

The lack of consensus among reported gene signature subsets (GSSs) in multi-gene biomarker discovery studies is often a concern for researchers and clinicians. Subsequently, it discourages larger scale prospective studies, prevents the translation of such knowledge into a practical clinical setting and ultimately hinders the progress of the field of biomarker-based disease classification, prognosis and prediction.

**Methods:**

We define all "gene identificators" (gIDs) as constituents of the entire potential disease biomarker space. For each gID in a GSS of interest ("tested GSS"/tGSS), our method counts the empirical frequency of gID co-occurrences/overlaps in other reference GSSs (rGSSs) and compares it with the expected frequency generated via implementation of a randomized sampling procedure. Comparison of the empirical frequency distribution (EFD) with the expected background frequency distribution (BFD) allows dichotomization of statistically novel (SN) and common (SC) gIDs within the tGSS.

**Results:**

We identify SN or SC biomarkers for tGSSs obtained from previous studies of high-grade serous ovarian cancer (HG-SOC) and breast cancer (BC). For each tGSS, the EFD of gID co-occurrences/overlaps with other rGSSs is characterized by scale and context-dependent Pareto-like frequency distribution function. Our results indicate that while independently there is little overlap between our tGSS with individual rGSSs, comparison of the EFD with BFD suggests that beyond a confidence threshold, tested gIDs become more common in rGSSs than expected. This validates the use of our tGSS as individual or combined prognostic factors. Our method identifies SN and SC genes of a 36-gene prognostic signature that stratify HG-SOC patients into subgroups with low, intermediate or high-risk of the disease outcome. Using 70 BC rGSSs, the method also predicted SN and SC BC prognostic genes from the tested obesity and IGF1 pathway GSSs.

**Conclusions:**

Our method provides a strategy that identify/predict within a tGSS of interest, gID subsets that are either SN or SC when compared to other rGSSs. Practically, our results suggest that there is a stronger association of the IGF1 signature genes with the 70 BC rGSSs, than for the obesity-associated signature. Furthermore, both SC and SN genes, in both signatures could be considered as perspective prognostic biomarkers of BCs that stratify the patients onto low or high risks of cancer development.

## Background

Current technology encourages the study of biological phenomena on a genome-wide scale. Technological platforms such as microarrays, next-generation sequencing, and mass spectrometry have resulted in generation of data on an unprecedented scale [1,2]. Inadvertently, the field of bioinformatics which includes high-performance cloud computing, adaptation of statistical methods, design of novel algorithms and generation of databases, play critical roles in the analysis of these massive and diverse datasets [3]. The variation in the type and amount of biological data, coupled with the fact that investigators may sometimes be confronted with a question that cannot be answered using current statistical techniques or algorithms [4], means that the field of statistical methods and algorithms is under constant refinement, adaptation and improvement [5].

Today, analysis of data from high-throughput experiments often yields a set of high-dimensional variable (HDV) list which typically represent a particular phenotype with respect to another. Such HDV lists commonly include signature lists of expressed genes, loci or proteins. Subsequent types of analysis to be performed on the gene list, depend greatly on the biological question an investigator is interested in. The work has been greatly simplified, partly due to the presence of many databases that were created, mostly in recent years [6-8]. The wealth of raw or curated, but nonetheless collated information in these databases is often critical in the subsequent analysis of gene (or other HDV) lists derived from these high-throughput experiments.

One of the most common analyses one could perform with a set of gene lists is an enrichment study of biological functions, processes or pathways with respect to a well-annotated reference gene list which commonly includes all the annotated genes in the genome. This analysis is commonly termed gene ontology analysis [9] which is based on simple statistical tests such as hypergeometric, binomial, or Chi-square tests [10]. These statistical tests could also be used if one is merely interested in whether one list of genes is similar to another, e.g. whether the gene products differentially expressed in human breast cancer (BC) are similar to the gene products differentially expressed in human ovarian cancer [8]. In addition, complementary methods such as Gene Set Enrichment Analysis (GSEA) allow the assessment of the relative relevance of one gene list of interest with reference to the expression differences of ranked genes between two phenotype cell classes of an organism [11].

Despite improvements in experimental technology and techniques, poor reproducibility and stability of results from independent but similar experiments can often hinder scientific discovery. These issues can arise due to many reasons such as small sample size [12,13], high-noise data [12-14], use of different technological platforms as well as poorly reported clinical or research protocols (different cohort classifications, treatment differences) [8,14-16]. In particular, small sample sizes, in combination with technical and biological noises, often complicate efforts to identify statistical differences of expression signals between many functionally important genes of distinct tumor subtypes or clinical groups. These limitations lead to bias in signature predictions and poor consistency. Inconsistency, divergence and poor overlap of many dozen of reported signatures suggest that our knowledge of nature and space dimensionality of tumor-associated genes and potential biomarkers is essentially incomplete [13,14,16]. Identification of potential biomarker space can reveal specific genetic patterns of cancer cells, for example, cell junctions in non-small cell lung cancer subtypes [17]. Our recent integrative studies of microarray gene expression profiles in lung adenocarcinoma (AC) versus normal adjacent lung tissue suggested that space dimensionality of potential biomarkers of lung adenocarcinoma (AC) is at least 2300 known genes [13]. Among these, hundreds of genes could be essential for disease-driving because they encode proteins containing mutagenesis sites, implying that these genes could be considered as relevant for diagnostic or prognostic applications [13]. In BC, the number of the genes that grade primary tumor by its aggressiveness is even larger; it consists of ~4000 microarray U133A detected genes [16].

To the best of our knowledge, current statistical analysis of matched lists mostly centres on comparison of two lists [18,19]. For example, independent studies of prostate cancer from two different countries may each yield a set of gene lists where genes are ranked by biological relevance, either by the magnitude of fold change, statistical significance of differential expression or other statistical measures. The comparison of the two gene lists based on robustness and stability can then be evaluated using a recently published method, which incorporates permutation studies and uses Canberra distance as the measure of dissimilarity [18]. However, methods which evaluate elements of one (new) list with reference to many other (known) lists is required in certain disciplines, including bioinformatics, genome-associated disease studies, medical statistics, epidemiology, ecology and authorship identification etc [20].

In cancer research, advances in high-throughput experimental techniques as well as statistical algorithms have resulted in the discovery of many gene signature sets (GSSs) which are representative of a particular cancer phenotype based on patient prognosis, tumor subtypes or other molecular features. However, it was reported that many of the GSSs generally do not show strong consensus for a given disease, even for more clinically homogeneous sub-groups of a disease (e.g. stage, tumor subtype) when independent patient cohorts are compared [12,13,21]. In BC, two well-known signatures which predict BC recurrence, comprising of 79 and 76 genes were derived independently in Amsterdam and Rotterdam cohorts respectively [15,22]. However, only three genes were found to be common. Nevertheless, it was stated that the rest of the unique genes could share similar pathways and be associated with similar mechanisms leading to the disease.

Recently, a systematic collection of gene expression signatures in cancers have been identified and collected in several databases [6-8]. For example, Abba et al. have collected 42 BC gene expression signatures in an effort to identify the most relevant BC biomarkers [23]. Comparison of these 42 signatures revealed limited or zero overlap between signatures. Specifically, comparison of the 3427 distinct gene symbols revealed that only 15 genes (*RRM2, MELK, MAD2L1, MYBL2, BIRC5, PTTG1, AURKA, PRC1, CKS2, CDCA8, MKI67, UBE2C, DUSP4, CENPF *and *CDC2*) are found in at least 10 signatures, which indicates the great disparity across gene signatures. The reasons for the disparity have been attributed to differences in clinical attributes of patients analysed which include ER status, stages, histological patterns, disease subtypes and treatment received by the patients.

Also, it is likely that each of the signatures represented only a partial picture of the heterogeneous and complex BC biology, which subsequently limits its potential for clinical implementation. The authors demonstrated that selection of several of these signatures having better consensus may provide more relevant and robust cancer biomarkers [23]. However, their strategy has a bias towards the larger size signatures and over-representation of cell cycle related genes. Other authors published a systematic review of gene expression signatures in colorectal cancer and identified 31 prognostic gene signatures [24]. It was reported that these gene lists, comprising a total of 1530 genes do not show great overlap, as there were only two common genes in four signatures, 10 common genes in three signatures, and 102 common genes in two signatures. It was stated by the authors that "the lack of gene overlap is generally interpreted as if each signature is a random sampling of a small subset of genes from a larger signature that represent the involved pathways [24]." However, without strong agreement and reproducibility of the gene signatures from independent studies, future large-scale prospective validation studies are unlikely to proceed, and may prove to be a major hindrance in achieving the desired goals of biomarker-based disease diagnosis, prognosis and prediction of therapeutic efficacy.

Many of the available biomarkers were generated in connection with biological functions of a given medical condition/disease. However, identity of the biomarkers for the same medical condition/disease could be quite different due to differences in the study design or analytical methods. Generally, it is very difficult to compare the GSSs due to such poorly-controlled variations in the process of discovery. However at times, it would be interesting to know how many times a particular gID in a newly derived signature has been reported in other known and reference GSSs (rGSSs) and whether the presence of the gID in these rGSSs occurs more frequently than expected by chance.

In our work, we address medical bioinformatics issues via computational intelligence, statistical analysis and computational simulation. Here, we proposed that a randomized sampling approach can provide a confidence indication of whether a gene is likely to be a common (and perhaps relatively more reliable) potential biomarker present in many other GSSs, or whether it is a relatively unique biomarker specific to a particular biological or disease state.

## Results

### Definition of novel or common biomarkers

**Figure 1:**
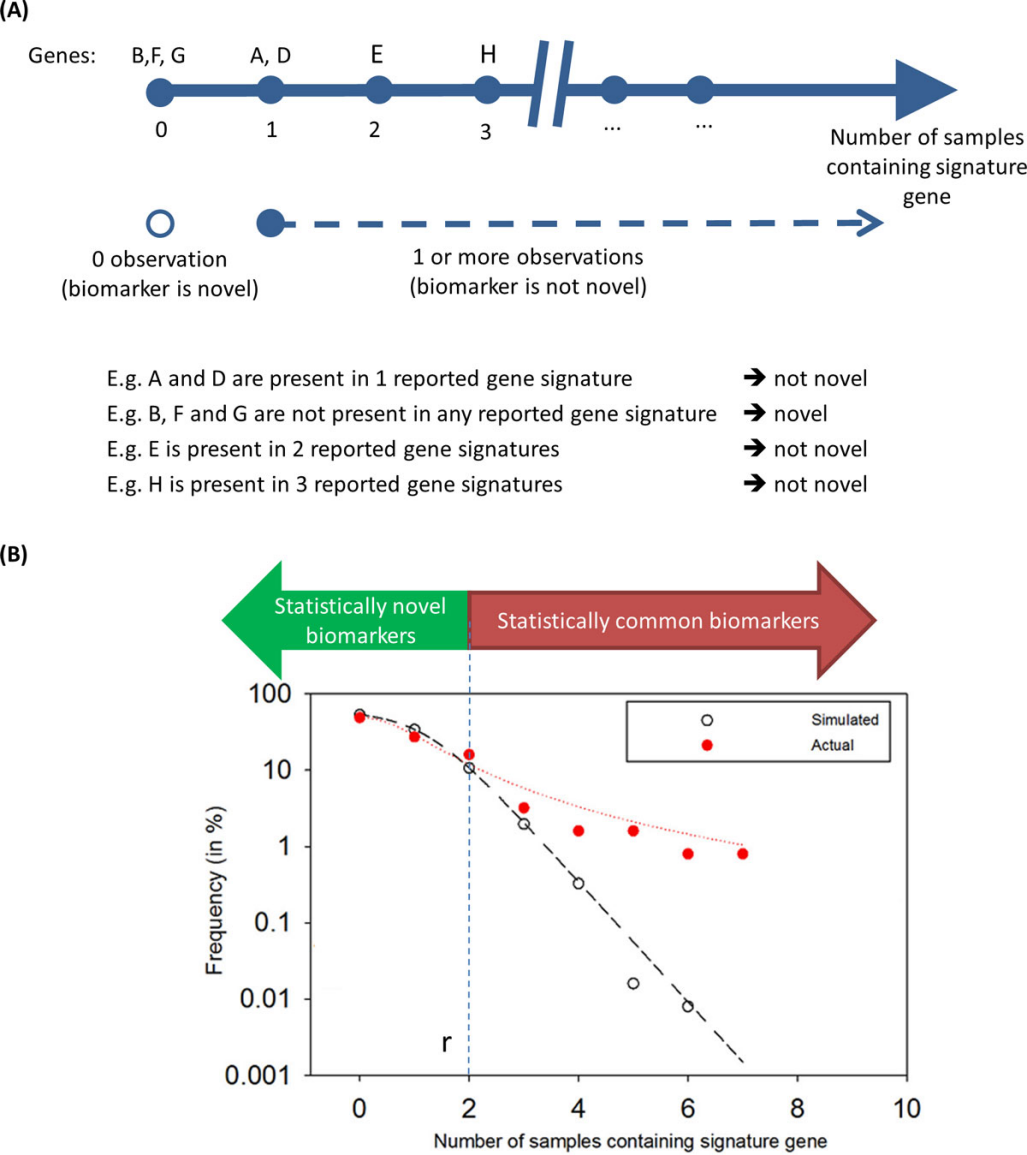
****Definition of novel or common biomarkers****. (A) Traditional definition of novel or common biomarkers. (B) Statistical definition of novel or common biomarkers. A further vertical dimension is extended which provides a statistical measure of whether the signature gene is considered "novel".

Traditional definition of novel biomarkers typically involves an absolute threshold value of 0, where only biomarkers which are not present in other published reference gene signature subsets (rGSSs) are defined as novel biomarkers (Figure 1A). In contrast, biomarkers which are present in at least one published rGSS are defined as "known" biomarkers. However, this definition does not account for the number of rGSSs under comparison, as well as ignore the variation in the number of biomarkers across rGSSs. For example, a biomarker present in only one of hundreds of rGSSs might be considered as a "statistically novel (SN) biomarker".

To address the above-mentioned issues, we first propose the construction of the observed frequency distribution function that describes the number of published rGSSs that contain each of the genes from the gene signature of interest. This function has a skewed shape with long right side tail. More precisely, the observed frequencies have the following characteristics in common: there are few frequent, and many rare events (clusters, interactions, co-occurrence etc). Such skewed functions are often observed in many natural and technological processes (the birth-death processes, biological evolution, interaction events in genome, transcriptome and proteome scales, artificial complex systems, physics phenomena, biological and social networks, industry incidences [25,26]. Sampling from such populations could be commonly fitted by the Pareto-like frequency distribution function, which is sample-size and context-dependent [25,26]. In practical applications, the left side of the observed skewed distribution could be enriched with 'admixture' events which consist of 'null' or 'background' additive noise events due to error measurements. In comparison with the Pareto-like frequency distribution function, such additional ('admixture') null or background frequency distribution (BFD) has a relatively shorter right-side tail. Such function could be described by the exponential distribution function or Poison distribution function. The identification of BFD and 'de-noising' of the empirical Pareto-like distribution function is a great challenge, specifically when sample sizes are relatively small [25,26].

We propose a simulation-based approach of random sampling to generate an expected BFD of the number of published rGSSs expected by chance to contain each of the genes from the GSS of interest (or "tested GSS"/tGSS) (Figure 1B). The method of random sampling to generate the BFD provides a fair basis of evaluating a significance measure of the empirical frequency of the number of the published rGSSs that contain each of the genes from the signature of interest. Implicitly, it takes into account the number of rGSSs under comparison, as well as taking into account the size variation across rGSSs. Effectively, it extends a vertical dimension (of expected and actual frequencies of the published rGSSs) to facilitate identification of statistically novel (SN) or common (SC) biomarkers (Figure 1B).

To discriminate between SN and SC biomarkers within tGSS derived from genome-scale data, we assume that the identity of individual element (potential biomarker represented by gene and/or probesets ID) is sufficiently well recorded as a potential biomarker, if that element appeared in the other lists more than r times (Figure 1B). With this assumption, we can compare the null BFD with actual empirical frequency distribution (EFD) and at the given confidence level, estimate a critical cut-off value of signature co-occurrences which subsequently allows us to discriminate biomarkers as SN or SC.

### Description of method

**Figure 2:**
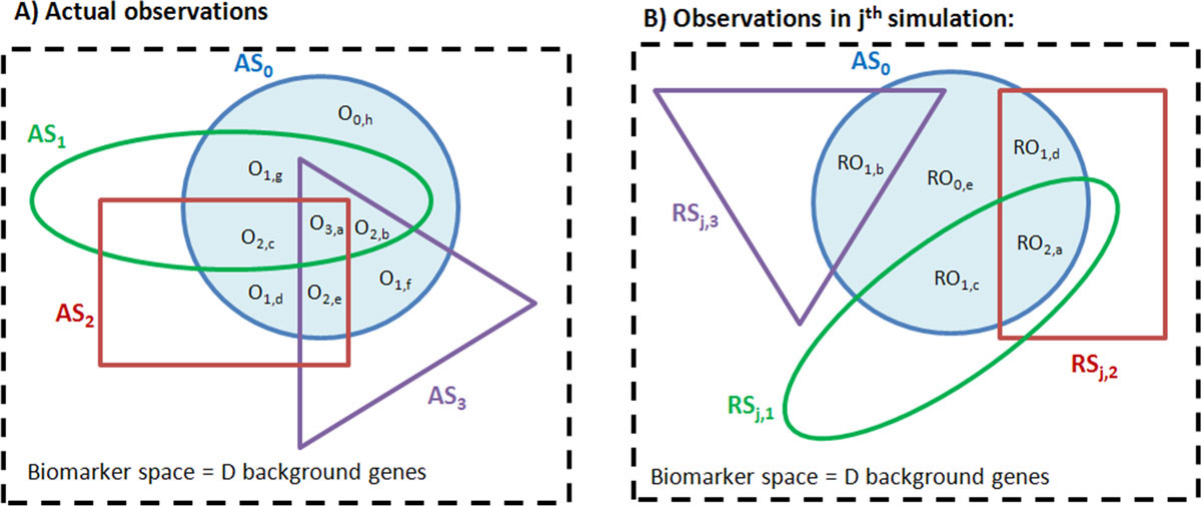
**Schema of gene list comparison with other defined sets**. (A) Actual observations of gene lists overlap between single list of interest (AS**_0_**) with other defined sets. (B) Observations of gene lists overlap in a simulation where other defined gene sets are randomly and independently sampled without replacement. AS and RS denote actual and random set respectively. O**_m _**and RO**_m _**denotes overlap segments and random overlap segments respectively. Blue solid circle represents our gene list of interest (AS**_0_**). Green oval, red rectangle and yellow triangle represent 3 other defined set of genes with sizes |AS**_i = 1_**|, |AS**_i = 2_**|, |AS**_i = 3_**| respectively.

An illustration of our proposed methodology is shown in Figure 2. We first define a background gene list which is a superset of all gene signatures. Appropriately, a background gene list could be defined as all human gene symbols or a subset of genes such as only those represented on a particular microarray platform. Genes in signatures which are not in the background gene list are removed. For each gene in a newly derived gene signature (AS_0_), the number of co-occurrences in M other published rGSSs is counted (AS_i = 1,2,3...M_) (Figure 2A). The EFD of number of genes for each co-occurrence events with the published rGSSs can be counted and plotted.

To generate the null BFD, several rounds of simulation are performed. In each simulation, random gene lists RS_i = 1,2,3...M _of equal sizes (where |RS_j,i_| = |AS_i_| for gene lists i = 1,2,3...M) are generated from the background gene list, without replacement within each simulation and with replacement between two simulations (Figure 2B). The number of co-occurrences of overlap between genes in AS_0 _with RS_i = 1,2,3...M _are counted in each simulation, and summed across all simulations. Subsequently, a null BFD of the number of gID matches with the random gene lists can be generated, which represents the expected frequency distribution of gID matches.

### Effect of background gene list

We first performed studies on simulated data to establish the family of null distributions that may result from this analysis. Specifically, the effect of the size of the background gene list, i.e. the effect of biomarker space on the null BFD is of interest. The background gene list, D, is first assumed to comprise of 20,000 genes. We assume that our gene signature, AS_0 _contains 100 genes, i.e. |AS_0_| = 100. Next, we assume that 10 other well-defined gene signatures are reported in the literature, each containing an unequal number of genes, e.g. |AS_1_|=20, |AS_2_|=40, |AS_3_|=60, |AS_4_|=80, |AS_5_|=100, |AS_6_|=120, |AS_7_|=140, |AS_8_|=160, |AS_9_|=180 and |AS_10_|=200.

Subsequently, we performed 100 simulations where in each simulation, the actual gene sets (AS_i_) are simulated by sampling the same number of genes independently from D without replacement. The expected frequency of observed co-occurrences of each gene in our gene signature can be determined.

**Figure 3:**
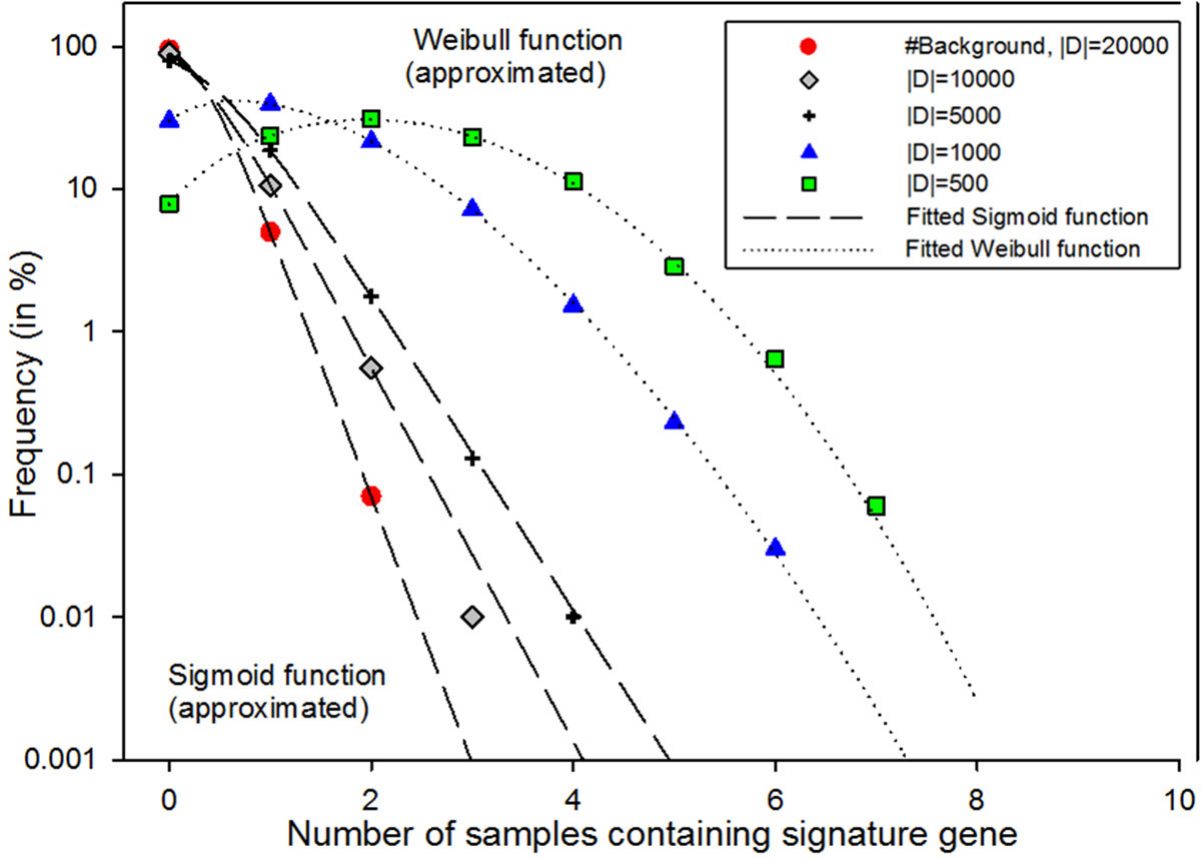
**Family of null frequency distribution of expected co-occurrences of our signature genes with other signatures**. The horizontal axis represents the number of samples that contain the gene from our signature of interest. The dotted lines represent the fitted curves of Weibull function whereas the dashed lines represent the fitted curves of Sigmoid function

Similar analyses are performed by repeating the procedures with reduced background gene lists (|D| = 10000, 5000, 1000, 500 genes). The effect of the size of the background gene lists can be observed in Figure 3. In addition, the expected null BFD can be approximated and fitted using Weibull or Sigmoid functions.

### Analysis of 36-genes prognostic signature for epithelial ovarian cancer

In our previous work [27], we studied expression and clinical data from patients diagnosed with high-grade serous ovarian cancer, and subsequently identified a 36-mRNA signature (assigned as the tGSS in this analysis) which stratifies patients into subgroups with very distinct and varied overall survival rates: low, intermediate or high-risk, with 5 year overall survival rates of 65%, 20% and 10% respectively. In addition to its prognostic significance, the 36-mRNA signature is also predictive of patients' response to chemo-therapy.

We compared our 36-gene tGSS with other published rGSSs of ovarian cancer. From the literature, we collected 63 rGSSs which were previously reported to show associations with survival, disease subtype, chemo-sensitivity, disease detection, development, progression or recurrence (Additional file 1). We restrict our analysis to official gene symbols present in the background gene set (RefGene version 30^th ^November 2012). After pre-processing of the gene symbols, each of the 63 rGSSs contain between 1 to 966 gene symbols. Subsequently, via 100 independent simulations, we generated a null BFD of overlap between our tGSS (comprising of 36 genes) with the randomly generated lists of signatures. Additionally, to understand the effect of the number of simulations on the null BFDs, we performed 1000 and 10000 independent simulations.

**Figure 4:**
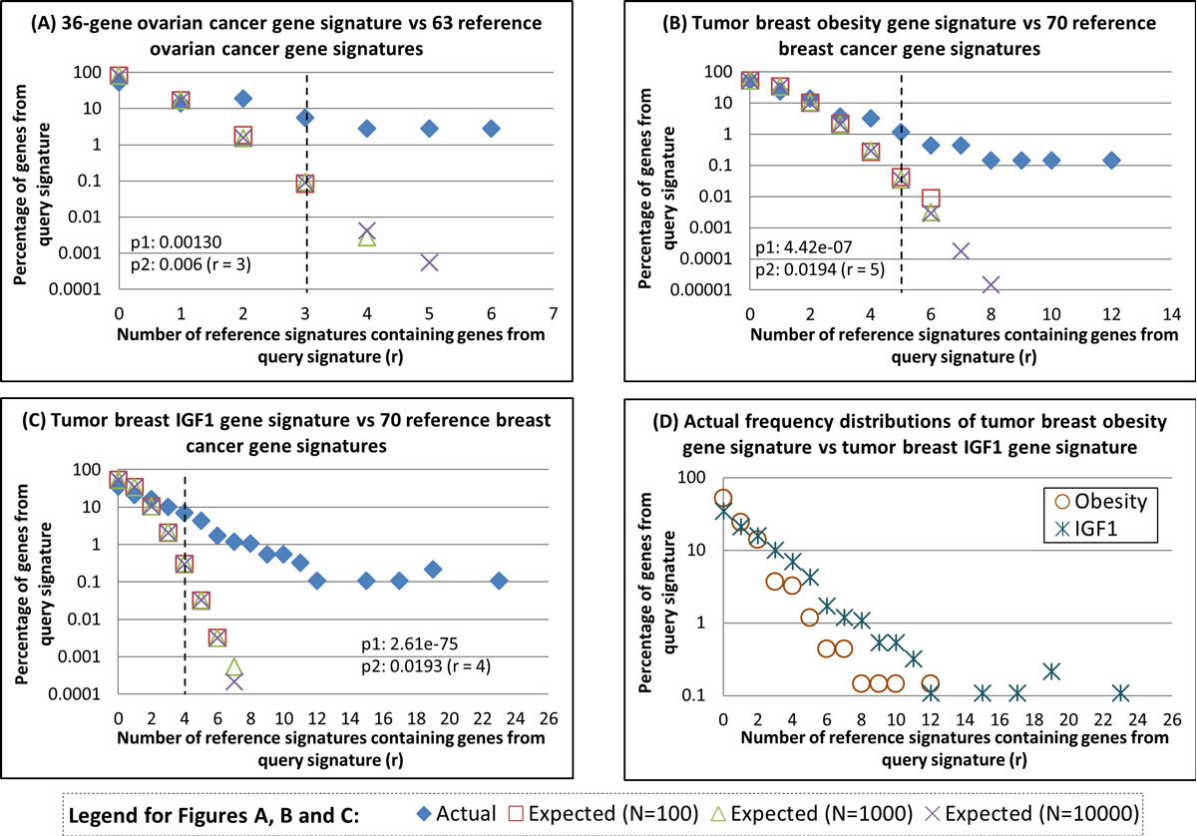
****Actual and expected frequency distribution of gene overlap from a query signature with other reference signatures****. Comparison of genes from (A) 36-gene ovarian cancer prognostic gene signature, (B) tumor breast obesity gene signature and (C) tumor breast IGF1 gene signature, with other reference gene signatures for that disease. (D) Comparison of the actual frequency distribution generated from tumor breast obesity (From B) and tumor breast IGF1 (From C). The expected frequency distributions were generated via performing N simulations, where N is 100, 1000 or 10000. The y-axis is log10 transformed. p1 denotes the two-sided p-value from Kolmogorov-Smirnov statistic which tests if the actual and expected (for N = 100) distribution are similar. p2 denotes the p-value that represents the significance of that threshold in dichotomizing statistically novel or common biomarkers from a GSS of interest.

The results of the comparison of our tGSS with other rGSSs are shown in Table 1 and Figure 4A (see also Additional file 3).

**Table 1:** Analysis of occurrence events for genes in the ovarian cancer prognostic gene signature set.

#Number of signatures with gene	*Expected Percentage (100 simulations)	Actual Percentage	Enrichment (actual/expected)	Number of genes from our signature	Genes from our signature
0	80.89	52.78	0.65	19	*POLA2, NCAPG2, PLAUR, FZD1, CCT2, DNMT1, PIK3R1, POLR2J, TGFBR2, VCL, NCAPD2, POLR2D, HGF, FGFR1, MIS12, ARPC1B, CD93, CDK4, NCAPH*
1	17.14	13.89	0.81	5	*MMP13, CBX3, CHEK1, LAMA4, TCP1*
2	1.89	19.44	10.29	7	*CDC6, CAV2, GNG12, CD44, MCM2, CALD1, CFD*
3	0.08	5.56	66.67	2	*CCL2, PDGFRA*
4	0.00	2.78	Not applicable	1	*TUBB*
5	0.00	2.78	Not applicable	1	*EDNRA*
6	0.00	2.78	Not applicable	1	*COL3A1*

Expected and actual frequency of co-occurrences of 36 genes in our prognostic signature with 60 other reference gene signature subsets. All signatures are ovarian cancer-related.

*The expected percentage from 1000 and 10000 simulations can be found in Additional file 3.

### Analysis of obesity and IGF1 signatures in breast cancer

Recently, the link between obesity and its contribution to poorer disease outcome in BC has been reported. Creighton and Sada et al. studied the effects of obesity on primary breast tumor gene expression and their results revealed an obesity-associated cancer transcriptional signature of 662 genes (assigned as the tGSS in this analysis) [28]. After preprocessing, this tGSS contains 683 RefSeq gene symbols (Additional file 2). Subsequently, we investigate whether individual genes in this obesity-associated tGSS derived from BC tumors are significantly enriched among the 70 BC rGSSs which were previously reported to be associated with clinical observations such as response to chemotherapy, distant metastasis, ER-alpha status, tumor subtypes and grades, as well as clinical outcomes such as patient prognosis. The 70 rGSSs comprise 42 gene signatures reviewed by Abba et al. [23] as well as those manually curated by us (Additional file 2). The background gene list consists of all RefGene symbols downloaded from UCSC Genome Browser on 30^th ^November 2012 and any gene that is not found within the background gene list is excluded from subsequent analysis.

As described in the methods, we performed 100 independent simulations and the enrichment of the genes in rGSSs relative to random expectation are shown in Figure 4B (Additional file 4). Overall, our results indicated that a large proportion of the obesity-associated tGSS genes have been reported as biomarkers in more BC rGSSs than expected from random simulations. For instance, based on random simulations, only 13.0% of the obesity-associated signature is expected to be found in at least 2 other rGSSs. However, 161 of 683 (23.6%) genes from the obesity-associated tGSS are actually found in at least 2 other rGSSs.

Also, it has been suggested that the link between obesity and BC outcome could be due to increased endocrine signaling involving insulin and insulin-like growth factors (IGFs) [28]. Specifically IGF1, whose expression is elevated in human breast cancer [29], is known to increase breast cancer cell growth and invasion [30]. Furthermore, activation of IGF1 pathway is correlated with early recurrence and decreased relapse-free survival [31].

Therefore, in subsequent analysis, we studied an IGF1 pathway gene signature (assigned as the tGSS in this analysis) whose genes were differentially expressed in MCF-7 BC cell line after IGF1 stimulation and which were correlated with several poor prognostic factors and disease outcome in patients with BC [32]. This IGF1 pathway tGSS contains 925 genes after gene identifier conversion and preprocessing. Similar analyses were performed for this tGSS with respect to the 70 other BC rGSSs and the results are shown in Figure 4C (Additional file 5). Similar to the obesity-associated tGSS, our results indicated that genes belonging to the IGF1-tGSS are generally found to co-occur in more rGSSs than expected from random simulations. Specifically, 43.6% of the IGF1-signature genes are found in at least 2 rGSSs, which is 3.3 times more than expected.

When we compared the EFDs for both the obesity-associated tGSS and IGF1 pathway tGSS, results suggested that there is a stronger association of the IGF1 pathway tGSS's genes with the 70 BC rGSSs than observed for the obesity-associated tGSS's genes with the 70 BC rGSSs (Figure 4D).

Next, we studied both the obesity and IGF1 tGSSs of BC with respect to the MAPK signalling pathway. From the KEGG database, the MAPK signalling pathway comprises of 267 genes, which included a list of 31 genes (*ATF2, CHUK, DDIT3, DUSP4, DUSP5, DUSP6, DUSP8, FGF13, FGFR2, FGFR4, FLNA, FOS, GADD45G, HRAS, IKBKB, IL1R1, MAP2K4, MAP2K6, MAP3K8, MAP4K3, MAPK9, MAPKAPK5, MAX, PAK2, PPM1B, PPP3R1, RAPGEF2, RASA1, RPS6KA3, RPS6KA4 *and *SOS1*) which are present in either or both of obesity-associated tGSS and the IGF1 pathway tGSS (Table 2). Subject to a minimum threshold of four rGSSs, genes present in at least one of the obesity or IGF1 tGSSs could be considered as SC gIDs. On the other hand, genes in either the obesity or IGF1-associated tGSSs that occur in three or less rGSSs are termed SN gIDs (Table 2). Typically, such genes which individually occur less frequently in other rGSSs are not easily interpreted with respect to their association with disease pathways or phenotypes. On the other hand, our method identifies many dozen genes of MAPK signalling pathway which have not been considered as a common BC signature in context of their functional association of obesity, IGF1 pathway and BC.

**Table 2:** Occurrence of MAP kinases signalling pathway genes in the studied breast tissue/cell signatures.

Gene Symbol	In obesity signature? (Yes/No)	In IGF1 signature? (Yes/No)	Number of reference signatures containing gene (out of 70)	Statistically novel (SN) or common (SC)
*ATF2*	Y	N	0	SN
*DUSP8*	N	Y	0	SN
*GADD45G*	Y	N	0	SN
*HRAS*	N	Y	0	SN
*IKBKB*	Y	N	0	SN
*MAP4K3*	Y	N	0	SN
*MAPK9*	N	Y	0	SN
*MAPKAPK5*	N	Y	0	SN
*MAX*	Y	N	0	SN
*PPM1B*	Y	N	0	SN
*PPP3R1*	N	Y	0	SN
*RAPGEF2*	Y	N	0	SN
*RPS6KA4*	N	Y	0	SN
*SOS1*	Y	N	0	SN
*CHUK*	N	Y	1	SN
*FGF13*	N	Y	1	SN
*FGFR2*	N	Y	1	SN
*MAP2K6*	N	Y	1	SN
*PAK2*	Y	N	1	SN
*RASA1*	Y	N	1	SN
*RPS6KA3*	N	Y	1	SN
*DDIT3*	N	Y	2	SN
*FLNA*	N	Y	2	SN
*IL1R1*	N	Y	2	SN
*MAP2K4*	Y	N	2	SN
*FGFR4*	Y	N	3	SN
*DUSP5*	N	Y	4	SC
*MAP3K8*	N	Y	4	SC
*DUSP6*	Y	Y	8	SC
*FOS*	N	Y	8	SC
*DUSP4*	Y	Y	12	SC

## Discussion

In this work, we developed a method for the identification of gene subsets that are statistically novel (SN) or common (SC) in a newly defined signature of interest when compared to other (known) reference gene signature sets (rGSSs) that are representative of a medical condition (e.g., breast cancer).

**Figure 5:**
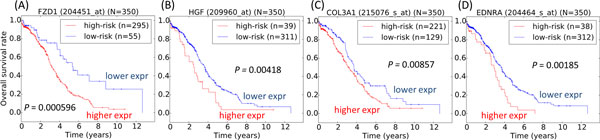
**Classification of high-grade serous ovarian cancer patients**. The patients diagnosed with high-grade serous ovarian cancer were classified using a data-driven method for statistically novel biomarkers (A) ***FZD1 ***and (B) ***HGF ***and common biomarkers (C) ***COL3A1 ***and (D) ***EDNRA***. Log-rank tests were used to assess the survival statistical significance of the two patient subgroups. Expr: expression.

We first provide an application of our methodology to one of our gene signature previously identified for the prognosis of patients diagnosed with high-grade serous ovarian cancer [27]. Our prognostic signature comprise of 36 mRNA genes (Signature#1 of Additional file 1). For this analysis, we manually curate 63 gene sets from the literature which were reported to show associations with survival, disease subtype, chemo-sensitivity, disease detection, development, progression or recurrence (Additional file 1). Our analysis revealed that more than 50% of the genes in our 36-gene prognostic signature were not previously considered as potential biomarkers in any of the curated publications (Table 1). These genes include *ARPC1B, CCT2, CD93, CDK4, DNMT1, FGFR1, FZD1, HGF, MIS12, NCAPD2, NCAPG2, NCAPH, PIK3R1, PLAUR, POLA2, POLR2D, POLR2J, TGFBR2 *and *VCL*. Despite the absence of these genes in currently known gene sets associated with the different aspects of ovarian cancers, some of these were nonetheless shown to play critical roles in cancer development and progression. For instance, it was shown that the majority of epithelial ovarian cancer tumors exhibited positive staining for *FZD1 *[33], and it was associated with chemo-resistance via the Wnt/Beta-catenin pathway [34,35]. Similarly for *HGF*, deregulated *HGF/MET *signaling is a common hallmark of many tumors and is associated with various aspects of tumor progression [36]. Furthermore, elevated *HGF *serum levels could predict poor prognosis in advanced ovarian cancers [37]. To the best of our knowledge, although *FZD1 *and *HGF *have not been reported as high-confidence biomarkers in ovarian cancer, their values as prognostic markers should not be neglected as is often the case. In fact in our previous analysis of high-grade serous ovarian cancer patients, both *FZD1 *and *HGF *exhibited significant prognostic properties (Figure 5A-B).

On the other hand, genes such as *CCL2, PDGFRA, TUBB, COL3A1 *and *EDNRA *have previously been identified as relevant biomarkers in at least 3 other published gene sets (Table 1). For instance, *EDNRA*, either independently or in combination with other biomarkers, was reported to be able to predict benign and malignant tumors from borderline tumors [38], tumor subtype classification [39,40] and classification of tumors related to cell plasticity [41]. *COL3A1 *is also a commonly studied gene in ovarian cancer, where it was revealed that it is one of the most expressed proteins in advanced relative to local ovarian adenocarcinoma [42], and that its expression was observed to be higher in platinum-resistant relative to platinum-sensitive cells [43]. Its use as a biomarker in prediction of chemo-sensitivity or platinum-sensitivity [43,44], determination of tumor subtype [39] or molecular subtype [40], and classification of tumors related to cell plasticity [41] was previously reported. Our data also indicated that both *EDNRA *and *COL3A1 *showed significant prognostic properties in patients diagnosed with high-grade serous ovarian cancer (Figure 5C-D).

In fact, our signature was originally derived for patient prognosis and prediction of chemo-sensitivity. In this analysis, we further found that certain genes in our 36-gene signature could be also relevant in tumor subtype classification. Also, we show that survival curves of several of our biomarkers show significance in patient stratification, regardless of whether they have been or not been reported in previous publications of ovarian cancer (Figure 5).

Next, we also studied two BC signatures from two published research reports (Signatures#1-2 of Additional file 2). The first signature was derived from differential expression analysis of obese versus non-obese BC patients [28]. The second signature was derived from differentially regulated genes in MCF-7 cells after IGF1 stimulation [32]. Independent comparison of these two signatures with the 70 BC rGSSs revealed that a larger proportion of genes from the IGF1 pathway gene signature were more likely to be represented in the rGSSs when compared to expectations from random simulation (Figure 4D, Additional files 3, 4). This might suggest a tighter association between the IGF1 signaling pathway with clinical observations such as response to chemotherapy, distant metastasis, ER-alpha status, tumor subtypes and grades, as well as clinical outcomes such as patient prognosis. In contrast, while we showed slight association between the obesity-associated gene signature with the rGSSs (Figure 4B,Additional file 4), this association appear to be weaker when compared to that of the IGF1 signaling pathway (Figure 4C, Additional file 5). This seems to suggest that the probable effects of obesity on cancer association could be less specific or directed, when compared to that of the IGF1 signaling pathway. Our method allows us to quantify a measure of associations between obesity, IGF1 pathway and BC signatures, as well as predict potentially SC and SN therapeutic target genes.

**Figure 6:**
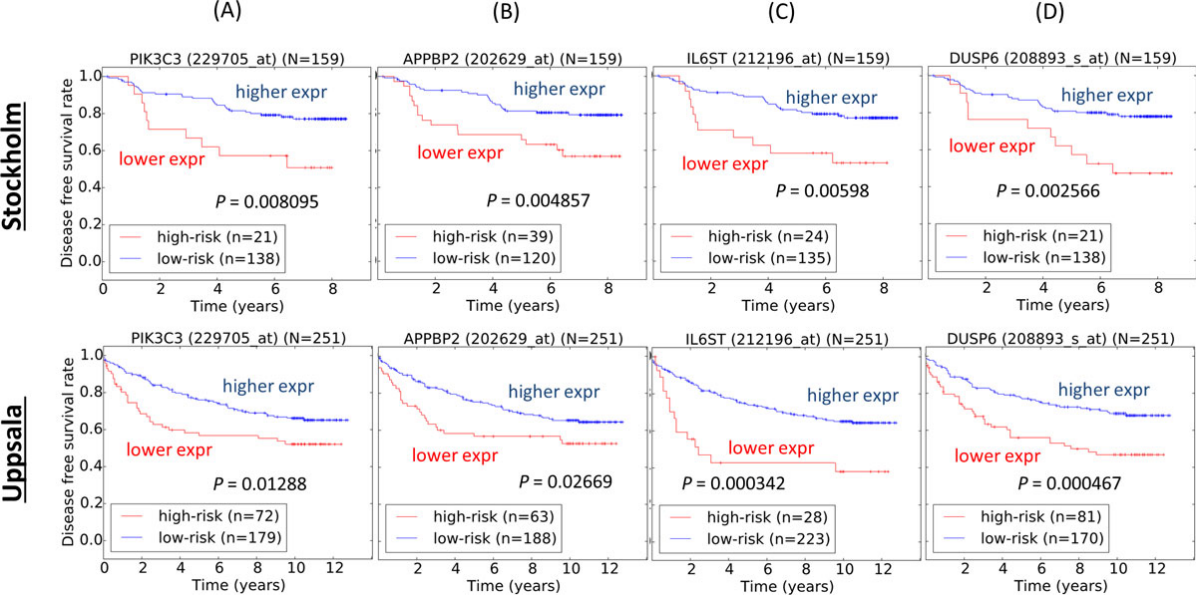
**Classification of breast cancer patients for both Stockholm and Uppsala patient cohort**. The patients diagnosed with breast cancer were classified using data-driven method for statistically novel biomarkers (A) ***PIK3C3 ***and (B) ***APPBP2 ***and common biomarkers (C) ***IL6ST ***and (D) ***DUSP6***. Top panel: Stockholm breast cancer patient cohort, Bottom panel: Uppsala breast cancer patient cohort. Log-rank tests were used to assess the survival statistical significance of the two patient subgroups. Expr: expression.

Additionally, using our published data-driven prognostic analytical method [45], we studied the survival significance of SN gIDs (*PIK3C3 *and *APPBP2*) and SC gIDs (*IL6ST *and *DUSP6*) of BC (Figure 6). Expression levels of SC gIDs such as *IL6ST *and *DUSP6*, which were found in 7 and 8 BC rGSSs respectively (Additional files 4 and 5), were found to be able to stratify both Stockholm and Uppsala BC patient cohorts into two survival significant subgroups via the data-driven grouping method (Figures 6C-D). Genes that were less studied and less represented in other rGSSs were traditionally neglected in terms of their prognostic capability. However, we show that expression levels of genes such as *PIK3C3 *and *APPBP2*, which were not found in any of the 70 BC rGSSs (Additional files 4 and 5), could also stratify both Stockholm and Uppsala patient cohort into two survival significant subgroups (Figures 6A-B).

While our general schema allows us to uncover the null background frequency distribution (BFD) of expected co-occurrences of our tSS's genes with other well-defined rGSSs, the specific use of this approach requires a careful formulation of the null hypothesis. Therefore, the background gene set D needs to be judiciously defined. In our example studies of BC and HG-SOC, we have defined the background gene set D as all annotated RefSeq gene symbols. However, sometimes it may be necessary to restrict the set of background gene list D to the list of genes that are tested, e.g. probesets on a microarray platform. The effect of a smaller background gene set D is non-trivial. Our simulated experiments on reduced background gene sets (|D| = 20000, 10000, 5000, 1000, 500) clearly revealed the rightward shift of the null BFD as the background gene set becomes more restricted (Figure 3). The interpretation of the actual empirical frequency distribution (EFD) and null BFD is therefore context-dependent and depends on the selection of the background gene list and other rGSSs. Therefore, the selection of the background set is non-trivial, and should generally be guided by the design of the experiments or formulation of the hypotheses.

We also studied the effect of the number of simulations on the null BFD. For each of the three tGSSs analysed, we generated null BFD from 100, 1000 or 10000 random and independent simulations (see Methods). Our results show that generally, 100 simulations are sufficient to uncover the structure and shape of the null BFD, which is representative of the percentage of tGSS that is expected to be found in the rGSSs at various levels of overlap counts. Increasing the number of simulations (1000 or 10000) might increase the sensitivity of our method in detecting random genes that likely appear in more rGSSs, but this is at the expense of higher computational cost.

Finally, in order to obtain a meaningful expected null BFD, the use of the method requires a considerable size of the signature of interest (|AS_0_|) and the number of other well-defined rGSSs (M). Therefore, the rapidly growing number of databases as well as the accumulating wealth at databases including but not limited to Gene Expression Omnibus (GEO) [6], ArrayExpress [7], GeneSigDB [8], meant that analysis of a newly generated gene lists with these previously defined gene sets could become more feasible. Certainly, the selection of the previously defined gene sets from the public repositories or literatures requires careful consideration as the eventual null BFD is representative of a scenario subject to the assumptions and conditions.

## Conclusions

The accumulating wealth of molecular signatures in publications and public repositories for a wide range of disease types and experimental conditions is a useful resource for any researcher. Importantly, it allows projection of a newly-derived GSS onto published, defined and well-annotated GSSs and subsequently identifies individual gIDs that are also independently associated with another feature. Our randomized sampling approach allows the generation of a null background frequency distribution, which could be used to identify a preliminary co-occurrence threshold and subsequently select subsets of the signature genes as statistically unique or significantly associated with another molecular feature. We have successfully applied our method to expression microarray and clinical data. In particular, our results suggest that there is a stronger association of the IGF1 signature genes with 70 BC rGSSs, than for the obesity-associated signature. Also, both SN and SC gIDs could be considered as perspective prognostic biomarkers of BCs. Our method identifies many dozen genes which have not been considered in context of their functional association of obesity and BC. In particular, our analysis suggests a close association of many genes expressed in MAPK pathways with obesity, IGF1 and BC pathways. We propose that the method reported in this study could also be applicable and of interest to researchers in other fields including but not limited to social science, epidemiology, linguistics, forensic analysis and internet networks.

## Methods

### Collection of gene sets

In our previous work, we identified 36 mRNA genes that are associated with overall survival and chemotherapeutic response of patients diagnosed with high-grade serous ovarian carcinoma [27]. This 36-gene signature is used as the testing signature of interest (or tGSS) for ovarian cancer (Signature#1 of Additional file 1). For breast cancer, we downloaded and analyzed two independent gene sets associated with obesity and IGF1 pathway respectively [28,32]. These two gene sets are defined as our testing signatures (or tGSS) of interest for BC (Signatures#1-2 of Additional file 2).

For the reference gene signature subsets (rGSSs), we compiled gene sets associated with BC and ovarian cancer from the literature. A total of 70 and 63 gene signature sets were collected for breast and ovarian cancers respectively. For BC gene sets, they were reported to predict clinical observations such as response to chemotherapy, distant metastasis, ER-alpha status, tumor subtypes and grades, as well as clinical outcomes such as patient prognosis (Additional file 2). The ovarian cancer gene sets were known to show associations with survival, disease subtype, chemo-sensitivity, disease detection, development, progression or recurrence (Additional file 1).

All gene accession identifiers are converted to official gene symbols.

### Definition of biomarker space

In this work, we defined the biomarker space as the official gene symbols represented on the RefGene database (downloaded from the UCSC genome browser on 30^th ^November 2012).

### Frequency distribution of actual gene occurrences in reference gene subsets

The visual representation as well as the notations of our methodology is shown in Figure 2. Assume that the entire biomarker space, D is well-defined and contains |D| genes. For example, a probable biomarker space could be defined by the number of reliable microarray probe-sets used for expression signal detection. Let AS_i _denote the actual set of genes, where i = 0 for a newly derived GSS (e.g. our gene signature or "testing GSS"/tGSS), and i = 1,2,3...M for M other reference GSSs (rGSSs), each containing a list of genes with size |AS_i_|>0. We assume that |AS_i_|<<|D| for i = 1,2,3...M.

Within our actual gene set AS_0_, the number of genes that are found in m other gene sets are denoted by observations, O_m = 1,2,3...M_, where

(1) $$O_{m=1,2,3\ldots M}=\sum_{s=segment}^{all\;segments\;with\;m\;observations}O_{m,s}$$

Also, the number of genes in our gene set (AS_0_) can be denoted by |AS_0_| where

(2) $$\left|AS_{0}\right|=\overset{M}{\underset{m=1}{\sum }}O_{m}=O_{1}+O_{2}+O_{3}+\ldots +O_{M}$$

The observed normalized frequency O_m = 1,2,3...M _is denoted by F_m = 1,2,3...m _where

(3) $$F_{m=1,2,3\ldots m}=\frac{O_{m}}{\left|AS_{0}\right|}$$

### Generation of random reference gene subsets via random sampling

For j^th ^of N simulations, RS_i,j _denotes a randomly and independently generated set of genes with size equal to AS_i_, i.e. |RS_j,i_| = |AS_i_| for gene lists i = 1,2,3...M. Each RS_j,i _is sampled randomly without replacement from the biomarker space D. Thus, the lists are produced by randomly sampling without replacement from biomarker space D, but the intensity of sampling might differ from list to list. In statistical literature such random collection was called a multiple record system [46].

The procedure is repeated N times, for N simulations.

Frequency distribution of expected gene occurrences in reference gene subsets

Next, the background model of the expected biomarker co-occurrence across other gene signatures is developed via sampling and simulations.

For j^th ^simulation, the number of genes in our gene set (AS_0_) that are also found in m gene sets RS_j,i = 1,2,3...M _is denoted by RO_j,m = 1,2,3,...M_, where

(4) $$RO_{j,m=1,2,3\ldots M}=\sum_{s=segment}^{all\;segments\;with\;m\;observations}RO_{j,m,s}$$

The expected normalized frequency of RO_m = 1,2,3,...M _after N simulations is

(5) $$EF_{m=1,2,3\ldots M}=\frac{\sum_{j=1}^{N}RO_{j,m}}{N\times \left|AS_{0}\right|}$$

### Comparison of the expected and actual frequency distribution of gene overlaps with other signatures

Subsequently, the observed and expected frequency of gene overlaps with other signatures can be seen, by comparing F_m _and EF_m _for m = 1,2,3,...M.

### Statistics of differences and identification of threshold stratifying statistically novel and common biomarkers of a signature

Initially, to assess the statistical differences between the observed normalized frequency (F) and expected normalized frequency (EF), we use Kolmogorov-Smirnov statistics to assess the statistical differences between the reverse cumulative frequency of F and EF.

When the difference between F and EF is significant, we aim to discriminate between statistically novel (SN) or common (SC) gIDs within a GSS of interest, by assuming that the biomarker is sufficiently well recorded as a potential marker if that biomarker appears in other rGSSs more than r times (Figure 1B).

For each discrete value r, where r ≥ 0, the p-value representing the significance of that threshold in stratifying SN or SC gIDs from a GSS of interest can be calculated from the ratio of the cumulative frequencies (expected with respect to actual) at that discrete value.

## List of abbreviations used

AC Adenocarcinoma

BC Breast cancer

BFD Background frequency distribution

EFD Empirical frequency distribution

gID Gene identificator

HDV High-dimensional variable

HG-SOC High-grade serous ovarian carcinoma

ID Identifier

IGF1 Insulin-like growth factor 1 (somatomedin C)

GEO Gene Expression Omnibus

GSS Gene signature subset

ncRNA Non-coding RNA

RefSeq Reference sequence

SC Statistically common

SN Statistically novel

rGSS Reference GSS

## Competing interests

The authors declare that they have no competing interests.

## Authors' contributions

VAK conceived and designed the study and together with OGS developed the algorithm of proposed methods. OGS implemented the methods. All authors analyzed the data, interpreted the results and wrote the manuscript.

## Supplementary Material

Additional file 1 **Gene sets compiled from published studies of ovarian cancer**. Click here for file

Additional file 3 **Analysis of occurrence events for genes in the ovarian cancer gene signature set**. Expected and actual frequency of co-occurrences of 36 genes in the ovarian cancer signature with other 60 gene signatures. All signatures are ovarian cancer-related. Click here for file

Additional file 2 **Gene sets compiled from published studies of breast cancer**. Click here for file

Additional file 4 **Analysis of occurrence events for genes in the tumor breast obesity gene signature set**. Expected and actual frequency of co-occurrences of 683 genes in the tumor breast obesity signature with other 70 gene signatures. All signatures are breast cancer-related. Click here for file

Additional file 5 **Analysis of occurrence events for genes in the tumor breast IGF1 gene signature set**. Expected and actual frequency of co-occurrences of 925 genes in the tumor breast IGF1 signature with other 70 gene signatures. All signatures are breast cancer-related. Click here for file

## Declarations

The publication costs for this article were funded by A*STAR, Singapore.

This article has been published as part of *BMC Genomics *Volume 16 Supplement 7, 2015: Selected articles from The International Conference on Intelligent Biology and Medicine (ICIBM) 2014: Genomics. The full contents of the supplement are available online at http://www.biomedcentral.com/bmcgenomics/supplements/16/S7.
